# Update on the Aetiopathogenesis of Sjögren Disease: From Interferon Signaling to Epithelial Dysfunction

**DOI:** 10.3390/jcm15051945

**Published:** 2026-03-04

**Authors:** Loïc Meudec, Gaetane Nocturne, Xavier Mariette

**Affiliations:** 1 Immuno-Rheumatology Department, Hôpital Bicêtre, Assistance Publique Hôpitaux de Paris (AP-HP), Université Paris-Saclay, 78 rue du Général Leclerc, 94275 Le Kremlin Bicêtre, France; loic.meudec@aphp.fr (L.M.); gaetane.nocturne@aphp.fr (G.N.); 2 Center for Immunology of Viral Infections and Autoimmune Diseases, Institut Nationale de la Santé et de la Rercherche Medicale (INSERM), UMR 1184, IDMIT, Université Paris Saclay, 78 rue du Général Leclerc, 94275 Le Kremlin Bicêtre, France

**Keywords:** Sjögren disease, interferon, B cells, epithelial cells

## Abstract

Sjögren disease (SjD) is a prototypical autoimmune disease whose management has long suffered from a limited understanding of its underlying pathophysiological mechanisms. However, major advances have been made over the past decade. The innate immune system is now recognized as playing a key role in the early stages of the disease, particularly through activation of interferon (IFN) pathways, driven in part by epithelial cells, which actively attract autoreactive lymphocytes. Furthermore, the mechanisms of B-cell activation in SjD are now better understood, notably with the recognition of BAFF (B-cell activating factor), a Tumor necrosis factor (TNF) family cytokine, whose production is highly dependent on type I and II IFN signaling. The involvement of other cell types, such as fibroblasts and T cells, has also been underlined. Significant progress has been achieved in elucidating lymphomagenesis, the most severe complication of SjD. Together, these advances provide a clearer picture of SjD pathogenesis and open avenues for the development of new targeted therapeutic strategies.

## 1. Introduction

Sjögren disease (SjD) is a systemic autoimmune disease characterized by a lymphocytic infiltration of the exocrine glands, particularly the salivary and lacrimal glands, so the term epithelitis is regularly used [1]. This infiltration leads to xerostomia and keratoconjunctivitis sicca. In addition, 40% of the patients develop systemic complications, such as renal, pulmonary, or neurological manifestations [1]. Moreover, approximately 5–10% of them develop lymphoma, the most serious complication of the disease.

**Figure 1:**
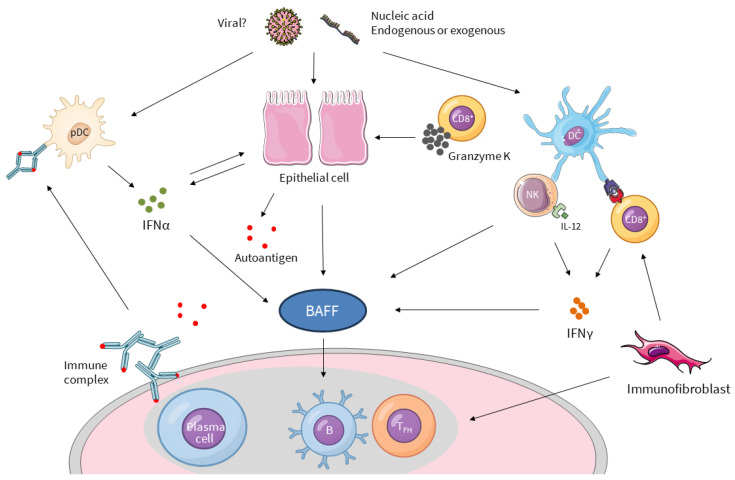
Current view of pathogenesis of SjD. Due to unknown stimuli that may be viral, the innate immune system is activated, leading to IFN production. IFNα is produced by plasmacytoid dendritic cells (pDC) and non-hematopoietic cells, including epithelial cells. NK cells and CD8+ T-cell activation lead to IFNγ secretion. IFN production results in the production of BAFF by various cell types, promoting B-cell activation in ectopic germinal centers, leading to autoantibody production and immune complexes. Immunofibroblasts contribute to the inflammation in the tissue by promoting ectopic germinal center formation and CD8+ T-cell activation. IFN and Granzyme K, produced by CD8+ T cells, also alter epithelial cells, leading to reduced exocrine function.

Translational research has led to major pathophysiological advances in three areas. First, the innate immune system plays a major role, highlighted by the interferon (IFN) signature observed in patients. Second, mechanisms underlying B-cell activation are better understood. Although several cytokines, including interleukin (IL)-21, are implicated, the Tumor Necrosis Factor (TNF) family cytokine B-cell activating factor (BAFF) appears to play a central role as a bridge between innate immunity and autoreactive B-cell activation, representing a promising therapeutic target. Advances have also clarified how B-cell hyperactivation contributes to lymphoma development in SjD. Finally, recent advances have shown the crucial role of epithelial cells, which are not merely passive targets but also contributors and, possibly, initiators of disease pathophysiology, as well as fibroblasts and CD8 T cells (Figure 1).

In this review, we focus on SjD pathogenesis. The clinical and therapeutic consequences of the pathogenesis of the disease are discussed in the same issue by Simon Bowman (Aspects of clinical disease beyond the dry eyes and mouth in Sjogren’s) and David Isenberg (Review of the use of biologic drugs in Sjogren’s), respectively.

## 2. Role of Genetics, Epigenetic, and Epitranscriptomic Regulations

### 2.1. Genetic Factors

Several arguments support a genetic predisposition to SjD. Although familial cases are rare, a family history of autoimmune diseases (AID) is common.

Genome-wide association studies (GWAS) conducted in SjD identified associations with major histocompatibility complex genes [2,3], such as HLA-DQA1*0501, HLA-DQB1*0201, HLA-DRB1*0301, and HLA-DRB1*0315, mostly in patients with autoantibodies [3,4,5].

Genetics also supports the role of IFN in the pathogenesis of the disease. Polymorphisms of *IRF5* (IFN regulatory factor 5) [6], a transcription factor involved in the IFN pathway, and *STAT4* (Signal Transducer and Activator of Transcription) [7], involved in type II IFN pathway and Th1 differentiation, have been found. These associations were confirmed by Lessard et al., coordinating a consortium of tens of thousands of patients [3]. Importantly, variants in the NFκB pathway regulators *TNFAIP3* (Tumor necrosis factor, alpha-induced protein) and *TNIP1* (TNFAIP3-interacting protein) have been described [3,8] as being associated with SjD onset, with an increased lymphoma burden regarding TNFAIP3 polymorphism [9].

Moreover, other polymorphisms have been described as involved in B-cell activation (*BLK*, *CXCR5*), JAK (Janus kinase)/STAT pathway (*TYK2*, *STAT1-4*), T-cell activation (*IL12A*, *HLA*), cell proliferation (*CD247*, *MIR146A*, *PRDM1*), and cell stress (*ATG5*, *CHMP6*) [3,7,10,11,12]. The association between SjD onset and *TNFAIP3*, *IRF5*, and *STAT4* has been confirmed in th Asian population, whose SjD incidence is increased, with notable differences regarding major histocompatibility complex (CMH) enrichment [2,13,14]. Polymorphism in *RBMS3*, involved in TGF-β regulation, has also been described in Asian populations [12].

These results support genetic predispositions involved in the immune response to inflammation in SjD, promoting the sustained inflammation observed in patients.

### 2.2. Epigenetics and Epitranscriptomics Modulations

Beyond genetics, epigenetics also contributes. It refers to DNA modifications that modulate gene expression without altering DNA sequences, including DNA methylation, histone modifications, and non-coding RNAs such as microRNAs (miRNAs) [15].

Hypomethylation of promoters of type I IFN-regulated genes has been shown in the blood of patients, leading to greater gene availability for transcription [16,17]. IFI44L (interferon-induced protein) was the most strongly affected IFN-induced gene [17], particularly in anti-Ro60/SSA positive patients [16].

miRNAs (micro-RNAs) are short non-coding RNAs that regulate messenger RNA (mRNA) post-transcriptionally [18]. Multiple studies have examined miRNA expression in the blood or salivary glands of SjD patients. Despite heterogeneous results, several miRNAs were associated with the disease (miR-146, miR-155, miR-181) [18,19].

Epitranscriptomic is another recently described layer of regulation [20]. Like DNA, RNA can be methylated, affecting its stability and translation into proteins. RNA methylation at the N6-methyladenosine (m^6^A) position can modulate inflammation and immune signaling. In the salivary gland, m^6^A acts as a negative feedback mechanism limiting inflammation in epithelial cells [21]. However, this control appears defective in SjD and may contribute to activation of IFN pathways in glandular tissue [21].

## 3. Activation of Innate Immunity and IFN Signature

IFNs are a family of cytokines first described in 1957 by Isaacs & Lindenmann for their antiviral activity, particularly against influenza [22]. Since then, IFNs have been implicated in several autoimmune diseases (lupus, myositis, scleroderma) [23], chronic viral diseases (HIV), and cancer. There are three IFN types: type 1, mainly IFNα (13 subtypes) and IFNβ, but also the less studied IFNε, IFNκ, and IFNω; type II (IFNγ); and type III, which includes IFNλ1, 2, and 3 (IL-29, IL-28A, and IL-28B, respectively). All three types signal through JAK/STAT. All types are involved in SjD, though distinguishing them was difficult until recently.

Several transcriptomic analyses of blood and salivary glands have shown increased expression of IFN-stimulated genes (ISG) in SjD [24,25,26,27]. Emamian et al. identified IFI35, MX1 (Myxoma resistance), OAS1 (oligoadenylate synthetase), IRF7, and OAS2 as highly differentially expressed in patients [24]. Interestingly, the expression of most ISGs correlated positively with anti-Ro60/SSA and anti-La/SSB titers, suggesting a link between innate immunity and B-cell activation.

The enriched ISG include the IFITM (interferon-induced transmembrane protein) family, involved in antiviral defense (*IFITM1*, *IFITM2*, and *IFITM3*) as well as Toll-like receptors (TLR) 8 and 9. The TLR pathway promotes IFN production by plasmacytoid dendritic cells (pDCs) and can be activated by immune complexes, viral particle cells, and infiltrating immune cells [28].

In the ASSESS (Assessment and Evolution of Systemic complications in primary Sjögren’s syndrome) cohort, elevated type I ISG *IFI27*, *IFI44*, and *OAS3* were associated with earlier disease onset and a higher ESSDAI (Eular Sjögren Syndrome Disease Activity index) scores (4 [2;8] versus 2 [1;7]) [29].

Several groups attempted to cluster patients by the IFN signature. Hall et al. found that more than 60% of patients showed an IFN signature and clustered into three groups: predominant type I, predominant type II, and mixed. Although clinical phenotypes were similar, the “predominantly type II” patients had higher focus scores [30]. Bodewes et al. also identified three clusters based on IFN signature: inactive IFN (*SMB9*, *NCOA7*, *TAP1*, *ISG20*, and *SP140)*, type I IFN (*IFI44*, *IFI44L*, *IFIT1*, *IFIT3*, and *Mx1)*, and type I + II IFN (*ZBP1*, *EIFAK2*, *IFIH1*, *PARP9*, and *GBP4).* Also, an active IFN signature correlated with higher activity in the ESSDAI biological domain [31]. A combined type I–type II signature was also observed in salivary glands [25].

The source of type I IFN production remains puzzling. While pDCs are present in salivary glands and produce large quantities in response to PAMPs or endogenous ligands [25], epithelial and other myeloid cells also contribute. Single-cell approach enables deeper cell characterization and indicates that dendritic cells, monocytes, and epithelial cells were the main producers of IFN, with the highest type I IFN score in endothelial cells [32]. Type II IFNs are also involved, likely appearing at a later stage through the activation of CD8+ T cells [33,34]. Type III IFNs have been characterized more recently. They are mainly produced by mucosal epithelial cells in barrier tissues and are enriched in SjD patients and sicca controls without SjD criteria. Interestingly, poly(I:C) induces type III IFN production by epithelial cells, highlighting microenvironment regulation [35].

The resulting hypothesis proposes initial innate immune cells activation by environmental, infectious, or endogenous triggers, leading to the production of IFNs by pDCs and epithelial cells (type I IFN) and by CD8 T cells and natural killer cells (type II IFN). Importantly, type I IFN and type II IFN induce BAFF production, driving B-cell activation [36]. Thus, BAFF appears to be at the crossroads of innate and adaptive immune system activation [37].

## 4. B-Cell Hyperactivation

### 4.1. The Role of BAFF

B-cell activation is a hallmark of SjD, as evidenced by the clinical and biological features observed in patients:

autoantibodies (rheumatoid factor, anti-Ro60/SSA, anti-La/SSB), polyclonal hypergammaglobulinemia, increased free light chains, and the higher risk of B-cell lymphomas [38].

The mechanisms underlying the hyperactivation are multiple, including cytokines, genetic, epigenetic, and environmental factors. The BAFF cytokine (B-cell activating factor of the TNF family) plays a central role, acting as a key link between innate immune activation and autoreactive B-cell survival. The BAFF cytokine was discovered in 1999 [39]. The same year, it was demonstrated by Mackay et al. that BAFF transgenic mice exhibited lupus and SjD-like features, including arthritis, glomerulonephritis, salivary gland infiltration, reduced salivary flow, autoantibodies (rheumatoid factor and anti-DNA), and an increased lymphoma risk [40]. Increased BAFF titers in SjD patients were further described by Mariette et al. in 2003 and correlate with autoantibody titers [41] and confirmed by many other studies.

**Figure 2:**
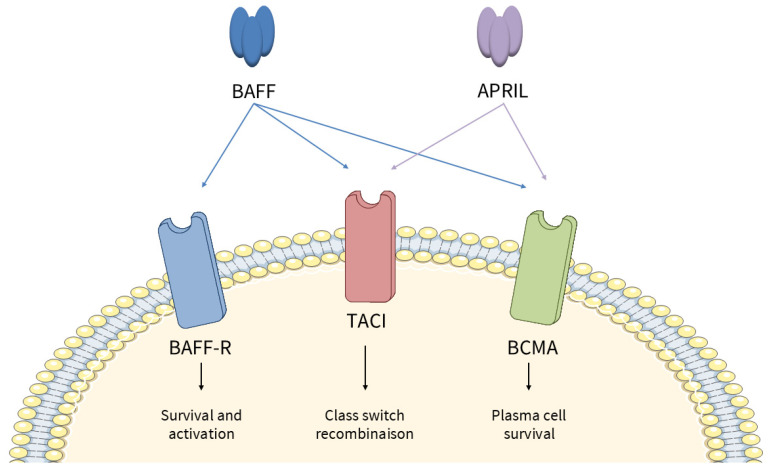
BAFF and its receptors. BAFF, a member of the TNF superfamily, regulates B-cell homeostasis through binding to three receptors: BAFF-R, TACI, and BCMA. BAFF-R is primarily involved in the survival and maintenance of mature naïve B cells, whereas TACI and BCMA are associated with later stages of B-cell differentiation, including class switch recombination and plasma cell survival. While BAFF interacts with the three receptors, APRIL interacts only with TACI and BCMA.

BAFF is expressed on the membrane of monocytes, macrophages, dendritic cells, and neutrophils and is secreted after proteolytic cleavage. In salivary glands of patients with SjD, BAFF could also be expressed by T cells [42] and B cells in an autocrine manner [43]. Non-hematopoietic cells can also produce BAFF, such as salivary gland epithelial cells, in response to IFN or Poly(I:C) [36]. Three BAFF receptors are expressed on B cells, whose expression and affinity vary with B-cell maturation: BAFF-R (BAFF receptor), TACI (transmembrane activator and calcium modulator and cyclophilin ligand interactor), and BCMA (B-cell maturation antigen) (Figure 2). BAFF-R promotes B-cell survival and maturation, while TACI is involved in class-switch recombination, and BCMA in the plasma cell long-term survival. BAFF acts as a BCR (B-cell Receptor) co-stimulator [44], enhancing B-cell proliferation, differentiation, and antibody secretion, and can induce class switching independently of CD40 ligand [45].

### 4.2. B-Cell Population

B cells play a central role in SjD pathogenesis, reflected by a strong B-cell signature in blood and tissue, including notably circulating plasmablasts and tissue plasma cells [46,47].

Differences in the distribution of memory B cells are observed in SjD between blood and salivary glands, with reduced circulating CD27+ memory B cells that could reflect their recruitment in the target tissue [48]. CyTOF (mass cytometry technique) analyses confirm this decrease in blood and enrichment at late differentiation stages in salivary glands [46].

Autoreactive plasma cells have been detected in salivary glands, although their origin remains unclear [49]. The presence of anti-Ro60/SSA and anti-La/SSB autoantibodies in glandular tissue supports their local production. Interestingly, infiltrating plasma cells display features of long-lived plasma cells: CD138 expression, non-proliferation, and Bcl-2 expression (anti-apoptotic protein). In addition, they localize near ductal and acinar epithelial cells expressing CXCL12, promoting the recruitment of lymphocytes [50]. Moreover, circulating plasmablasts are increased in SjD, particularly with anti-SSA autoantibodies [46].

Regulatory B cells (Breg) may also be involved. Breg produce IL-10, reducing Th1 T-cell proliferation and promoting regulatory T-cell (Treg) expansion [51,52]. IL-10 secretion ability of Breg appears preserved in SjD [53,54]. However, Breg function also depends on other cytokines, such as IL-35, an immunoregulatory cytokine composed of two heterodimers, IL-12p35 and EBI3. An imbalance between the pro-inflammatory IL-12 and IL-35 has been reported in patients, with reduced IL-35 and elevated IL-12 levels in SjD patients [55]. Moreover, higher IL-35 levels were associated with lower disease activity.

### 4.3. Autoantibodies

Autoantibodies are a central immunological feature of SjD. Anti-Ro/SSA antibodies are the most characteristic and are included in the current classification criteria. Ro60 is an intracellular RNA-binding protein involved in RNA quality control, promoting the degradation of misfolded RNA. Although anti-Ro60 antibodies are strongly associated with systemic manifestations and a type I interferon signature [30,31], a direct pathogenic role in disease onset has not been conclusively demonstrated.

Of note, about 30% of SjD patients lack anti-Ro60 autoantibodies [56], suggesting that additional immune mechanisms and other autoantibodies, some of which have only recently been described [57], may contribute to disease pathogenesis.

In addition, anti-Ro52 antibodies are commonly detected in SjD patients. Ro52/TRIM21 is an ISG encoding an E3 ubiquitin ligase involved in the ubiquitination process. Despite their low specificity for SjD, anti-Ro52 correlates with a higher IFN signature, with the strongest type I IFN profile in triple-positive patients for anti-Ro60, anti-Ro52, and anti-La [58].

### 4.4. Tertiary Lymphoid Organs

BAFF is not the only way of stimulating B cells in SjD. The CD40/CD40L axis plays an important role and may favor the formation of ectopic germinal centers in the salivary glands [59]. Soluble CD40L has been observed in SjD [60], with increased expression on T cells from patients and higher expression of CD40 on infiltrating B cells supporting this point [61]. The immune infiltrate in the salivary glands includes plasma cells, but also T cells, B cells, macrophages, follicular dendritic cells, dendritic cells, and plasmacytoid dendritic cells. Germinal centers similar to those seen in secondary lymphoid organs form in about a quarter of patients [62], making the salivary gland a tertiary lymphoid organ (TLO). TLOs, which arise from chronic inflammation, mirror secondary lymphoid organs in cellular composition, structure, and function and contribute to disease progression and increased lymphoma risk [63]. Their formation has recently been linked to the presence of fibroblasts [64].

### 4.5. Lymphomagenesis

**Figure 3:**
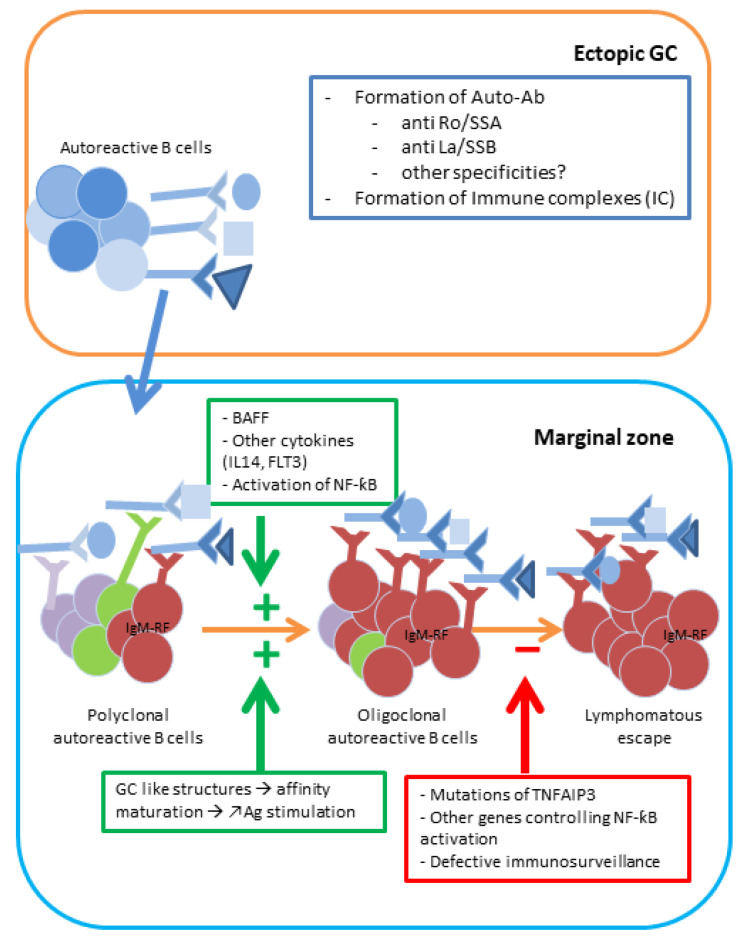
**Lymphomagenesis in SjD**. Lymphomagenesis in the course of SjD results from chronic B-cell stimulation within ectopic germinal centers. Autoreactive B cells with rheumatoid factor activity are continuously activated by immune complexes composed of autoantibodies and autoantigens, as well as by cytokines (notably BAFF), leading to sustained activation of the NFκB pathway. Polymorphisms affecting key regulators of B-cell activation (TNAIP3, NFκB) further contribute to an exaggerated inflammatory response, thereby promoting the emergence of lymphoma clones, predominantly within organs targeted by autoimmunity.

SjD carries the highest lymphoma risk among autoimmune diseases (10–15 fold) [38], representing the final stage of chronic autoreactive B-cell hyperactivation (Figure 3). About 5–10% of patients develop lymphoma, predominantly low-grade marginal zone (MALT) lymphomas (65%).

Lymphoma B cells in the context of SjD are autoimmune B cells continuously stimulated by autoantigens [65] and frequently rheumatoid factor B cells stimulated by the high level of immune complexes composed of autoantibodies complexed with autoantigens [66]. Lymphomagenesis sheds light on the chronic stimulation of autoreactive B-cell clones in a multi-step process. The chronic stimulation, combined with a favorable cytokine environment (BAFF) and defective inflammation control mechanisms (*TNFAIP3* mutation and NFκB pathway), favors malignant transformation [67]. These steps may guide the development of targeted therapies in SjD.

## 5. T-Cell Involvement

Lymphopenia is a common feature of SjD, but peripheral lymphocyte distribution is only slightly altered. However, T-cell function is impaired, with reduced T-cell proliferation upon anti-CD3 stimulation [68].

Within the salivary glands, most infiltrating T cells are CD4+ (70–80%), but an important role of CD8+ T cells has recently been highlighted [69]. These infiltrating T cells are activated, evidenced by increased HLA-DR and CD25 expression [68].

IL-7 is a major cytokine regulating T cells, playing a homeostatic role by promoting survival of naive and memory T cells expressing IL-7 receptor (CD127) [70]. Bikker et al. reported elevated IL-7 and CD127 expression in the salivary glands of patients [71], correlated with immune infiltration. The IL-7/IL-7R axis supports the formation of ectopic germinal centers in the salivary glands [72]. Moreover, a reciprocal activation loop between T cells and salivary gland epithelial cells (SGEC) has been demonstrated. IL-7 activates T cells in the glands, inducing IFN-γ production, which, in turn, activates SGEC [73].

Resident memory CD8 T cells have been identified in the SjD salivary gland, which produce granzyme B, contributing to the production of IFN-γ in the tissue [33,74]. Interestingly, these cells express CD103, which can be targeted, resulting in reduced glandular damage in mouse models [33]. Exhausted memory CD8+ T cells expressing Granzyme K have also been described recently in the salivary glands of patients [34,75]. Unlike Granzyme B, Granzyme K does not induce apoptosis but promotes IFN production by target cells. Notably, Granzyme K CD8+ T cells colocalize with acinar cells and may directly contribute to SGEC dysfunction.

Data regarding regulatory T cells (Tregs) in SjD are contradictory [76,77,78], partly due to variability in phenotypic markers used for their identification [79]. Christodoulou et al. showed that FoxP3+ T-cell infiltration was inversely correlated with the focus score [80].

## 6. Epithelial Cells, an Active Actor

SGECs are composed of acinar cells, which produce the components of saliva (acini), and ductal cells, forming the ducts that transport saliva to the oral cavity, ensuring exocrine function [81]. An alteration of SGEC function is observed in SjD, with morphological and functional alterations of the acini and immune infiltration around the ducts.

Epithelial abnormalities are found in various types of epithelial cells (bile ducts, renal tubules, etc.), supporting the term “autoimmune epithelitis” that has been used to describe the disease [82]. A selective loss of PRR4^+^CST3^+^WFDC2^−^ seromucous acinar cells has recently been identified using a single-cell approach [34]. SGECs express a higher level of HLA-DR in SjD, correlated with IFNα, as well as higher expression of adhesion and co-stimulatory molecules CD80 and CD86. In addition, SGECs from SjD patients also display senescence marker p16, suggesting increased cellular turnover [83].

Although the immune infiltrate is commonly considered responsible for dryness, the absence of immune infiltrate in about 20% of patients suggests specific epithelial abnormalities [56]. Intrinsic abnormalities of SGECs have been demonstrated using three-dimensional salivary gland organoid (SGO) culture systems, allowing the evaluation of SGECs in the absence of an inflammatory environment [61,62]. Even without immune cells, SjD-derived SGOs exhibit disease-specific hallmarks, including altered branch forming, persistent IFN signature, and an impaired cholinergic response [84]. These results show that SGECs undergo intrinsic and durable alterations that are not solely dependent on immune infiltration, challenging the notion that glandular dysfunction is exclusively secondary to inflammation.

The neuronal system could also be involved in glandular dysfunction and immune infiltration. Clinical neuropathy occurs in about 15% of patients [85], and neural abnormalities appear to play a particular role in ocular dryness. Notably, decreased corneal nerve density has been demonstrated in SjD compared with both healthy controls and individuals with idiopathic dry eye disease, which correlates with tear dysfunction [86]. Thus, dysimmune neurological abnormalities may contribute to glandular dysfunction. Interestingly, Aire-deficient mice, which develop T-cell-mediated autoimmune exocrinopathy, exhibit dry eye associated with reduced corneal nerve density. Of note, these mice also show decreased acetylcholinesterase activity [87].

In addition, reduced activity of the hypothalamic–pituitary–adrenal (HPA) axis, a key component of the peripheral neuroendocrine system, has been described in SjD along with estrogen deprivation [88]. Estrogen deficiency promotes increased expression of retinoblastoma-associated protein 48 (RbAP48). Interestingly, mice models that overexpress RbAP48 show oral and ocular dryness, immune infiltrate, and anti-SSA production [89]. Thus, neuro-immune interactions could contribute to epithelial impairment in SjD.

Acinar alteration also contributes to the development of a hyperosmolar environment in salivary gland ducts, promoting inflammation maintenance by driving epithelial-to-mesenchymal transition (EMT), extracellular matrix remodeling, and immune infiltration [90].

SGECs also play an active role in sustaining inflammation in SjD. SGECs promote B-cell survival and activation and can produce BAFF and IL-7 upon IFN and Poly(I:C) stimulation [91], establishing a dialogue with infiltrating B cells through secreted molecules and cellular contacts (CD40/CD40L) [73,91]. These pathways may represent targets for emerging therapies.

Stromal cells form the structural compartment of the gland and mainly include fibroblasts, as well as endothelial cells, nerve cells, and resident immune cells. Fibroblasts have recently gained attention in SjD and may actively contribute to inflammation maintenance and fibrosis. Immunofibroblasts are of particular interest due to their role in establishing tertiary lymphoid structures, thus supporting B-cell maturation in salivary glands [64,92]. Interestingly, immufibroblasts and CD8+ T cells colocalize within the gland, suggesting their interaction, which could be due to *C3*-*ITGB2* and *CXCL14*-*CXCR4* interactions [74].

## 7. Future Direction: Toward a Better Understanding of SjD Heterogeneity

SjD is a clinically heterogeneous disease, raising the question of the variable involvement of the molecular pathways mentioned above across patients. This heterogeneity is fundamental in understanding the disease. Numerous studies have sought to define patient subgroups to better assess prognosis and, ultimately, enable personalized treatment.

Several approaches have been proposed. Some rely solely on symptoms [93,94], while others use multiomic data, integrating genetic, epigenetic, transcriptomic, cytometric, and cytokinetic data. Using this latter approach, four clusters have been described as follows [95]: C1, characterized by a marked type I and type II IFN signature; C2, resembling healthy subjects; C3, combining an intermediate type I and type II IFN signature with B-cell hyperactivation; and C4, marked by a predominant type II IFN signature.

A middle-ground approach has been developed based on clinical and biological features accessible in routine practice. Three patient subgroups were identified: high systemic activity (HSA), low systemic activity with biological signs of B-cell hyperactivation (BALS), and low systemic activity but high functional burden (LSHS) [96]. Interestingly, these subgroups show different risks of progression. Patients without systemic activity but significant symptoms do not progress, in contrast to BALS. Within this subgroup, the presence of an IFN signature at baseline is associated with a higher risk of requiring immunosuppressive drugs and with a tendency toward an increased incidence of lymphoma [97].

## 8. Conclusions

Innate immunity is involved in the early stages of SjD: an environmental or endogenous trigger is likely to promote activation of the IFN system and epithelial cell inflammation in individuals with genetic risk factors, particularly those associated with an increased activation of the IFN pathways. These individuals are susceptible to enhanced immune activation, notably increased BAFF production, which supports the survival of autoreactive B cells. In some patients, continuous antigenic stimulation of autoreactive B cells leads to lymphomatous transformation through a multistep process that may include genetic alterations affecting the NFκB regulatory pathway, increased levels of immune complexes, and continuous stimulation of autoimmune B cells. B-cell activation is, therefore, a central feature of SjD pathophysiology, making B-cell-targeted therapies particularly promising.

Nevertheless, SjD does not rely solely on B cells. There is substantial evidence supporting the involvement of other cell types, including SGECs, several T-cell subtypes, and fibroblasts. Improved understanding of SjD pathophysiology has paved the way for the development of immunotherapies, which, we hope, will soon benefit patients.

## Data Availability

No new data were created or analyzed in this study. Data sharing is not applicable to this article.
